# Age Differences in Prefrontal Surface Area and Thickness in Middle Aged to Older Adults

**DOI:** 10.3389/fnagi.2015.00250

**Published:** 2016-01-19

**Authors:** Vonetta M. Dotson, Sarah M. Szymkowicz, Christopher N. Sozda, Joshua W. Kirton, Mackenzie L. Green, Andrew O’Shea, Molly E. McLaren, Stephen D. Anton, Todd M. Manini, Adam J. Woods

**Affiliations:** ^1^Department of Clinical and Health Psychology, University of FloridaGainesville, FL, USA; ^2^Department of Neuroscience, University of FloridaGainesville, FL, USA; ^3^Department of Aging & Geriatric Research, University of FloridaGainesville, FL, USA; ^4^Center for Cognitive Aging and Memory, Institute on Aging, University of FloridaGainesville, FL, USA

**Keywords:** adult age differences, cortical thinning, volumetrics, aging, MRI

## Abstract

Age is associated with reductions in surface area and cortical thickness, particularly in prefrontal regions. There is also evidence of greater thickness in some regions at older ages. Non-linear age effects in some studies suggest that age may continue to impact brain structure in later decades of life, but relatively few studies have examined the impact of age on brain structure within middle-aged to older adults. We investigated age differences in prefrontal surface area and cortical thickness in healthy adults between the ages of 51 and 81 years. Participants received a structural 3-Tesla magnetic resonance imaging scan. Based on *a priori* hypotheses, primary analyses focused on surface area and cortical thickness in the dorsolateral prefrontal cortex, anterior cingulate cortex, and orbitofrontal cortex. We also performed exploratory vertex-wise analyses of surface area and cortical thickness across the entire cortex. We found that older age was associated with smaller surface area in the dorsolateral prefrontal and orbitofrontal cortices but greater cortical thickness in the dorsolateral prefrontal and anterior cingulate cortices. Vertex-wise analyses revealed smaller surface area in primarily frontal regions at older ages, but no age effects were found for cortical thickness. Results suggest age is associated with reduced surface area but greater cortical thickness in prefrontal regions during later decades of life, and highlight the differential effects age has on regional surface area and cortical thickness.

## Introduction

Advancing age is associated with structural brain changes, even in the absence of dementia or other pathological conditions (Raz et al., 2007a). The frontal and temporal lobes are most affected, with many studies showing disproportionate changes in regions within the prefrontal cortex (Small et al., 2000; Liu et al., 2003; Resnick et al., 2003; Raz et al., 2004, 2010; Raz and Rodrigue, 2006; Driscoll et al., 2009; Fjell et al., 2009b; Spreng et al., 2010), including the anterior cingulate (ACC), dorsolateral prefrontal (DLPFC) and orbitofrontal (OFC) cortices. Much of our understanding of age differences and age changes in brain structure comes from volumetric studies, which typically show reductions in regional volumes with age, although increased volumes have also been reported (Salat et al., 2002).

Gray matter volume is a composite measure that is derived from both surface area and cortical thickness, separate measures that may be differentially affected by the aging process (Fleischman et al., 2010; Marsland et al., 2015), due, at least in part, to their differential relationship with genetic and environmental factors (Jefferson et al., 2007; Panizzon et al., 2009; Krishnadas et al., 2013). Individual differences in brain volumes may be more closely related to surface area rather than cortical thickness (Reuter et al., 2010; Winkler et al., 2010; Solana et al., 2012), while cortical thickness is thought to be more closely related to features of the cortex such as the organization of cortical layers and the size, number, and density of cell bodies in the neurons, as well as synaptic connections (Kabani et al., 2001; Du et al., 2007). Supporting the contention that surface area and cortical thickness are independent measures of brain structure are findings that the two measures are negatively correlated in older adults (Hogstrom et al., 2013; Fjell et al., 2014; Storsve et al., 2014).

Both cross-sectional and longitudinal studies have provided evidence for decreased surface area and cortical thinning at older ages (Salat et al., 2004; Preul et al., 2006; Fjell et al., 2009b; Ostby et al., 2009; Thambisetty et al., 2010; Lemaitre et al., 2012; Long et al., 2012; Solana et al., 2012; Hogstrom et al., 2013; van Velsen et al., 2013), with many showing disproportionate reductions in the prefrontal cortex (Salat et al., 2004; Thambisetty et al., 2010; Lemaitre et al., 2012). Older age is consistently associated with decreases in surface area, but the literature regarding age and cortical thickness is mixed, with some showing age-related decreases and others showing age-related increases. For example, in healthy adults age 18–91 years from the Alzheimer’s Disease Neuroimaging Initiative sample, most regions were found to be associated with cortical thinning at older ages, but a nonlinear effect of age was found for the rostral and caudal ACC, such that cortical thickness was greater after age 60 (Fjell et al., 2014). Increased thickness at older ages was postulated to be related to neuroplastic adaptation to increased environmental demands, given the ACC’s role in the allocation of attentional resources, or to a cross-sectional artifact since longitudinal increases in thickness were not observed in the ACC (Fjell et al., 2014). However, results from the Baltimore Longitudinal Study of Aging provide evidence of longitudinal increases in cortical thickness in multiple frontal, temporal, and parietal regions (Thambisetty et al., 2010) despite an overall pattern of decreased cortical thickness with age in most regions. Another study reported selective increases in cortical thickness in older adults who performed well on fluid intelligence measures (Fjell et al., 2006), suggesting that sample differences in cognitive abilities may contribute to the different findings across studies. The mechanisms underlying age-related increases in cortical thickness are unclear, but increases in low-grade inflammation with age may contribute, as inflammatory markers have been associated with cortical thickening (Sörös, 2010; Krishnadas et al., 2013).

Despite the accumulation of knowledge regarding age-related structural changes, differences in results across previous studies highlight the need for additional research to clarify the nature and timing of these changes. As the population continues to age, it is particularly important to complement studies that compare young and older adults with examinations of age differences within middle-age to older adults. Such studies will help elucidate the potentially progressive brain changes that occur in later decades of life. Longitudinal studies in middle-age to older adults, as well as cross-sectional lifespan studies, show non-linear structural brain changes (Sowell et al., 2003; Allen et al., 2005; Du et al., 2007; Curiati et al., 2009; Driscoll et al., 2009; Fjell et al., 2009b, 2014; Raz et al., 2010; Thambisetty et al., 2010; Schuff et al., 2012; Ziegler et al., 2012; Pfefferbaum et al., 2013; Taki et al., 2013), suggesting age differences may be apparent within middle aged to older samples, rather than only being evident when comparing young and older adults. Examining age differences in both surface area and cortical thickness within the same sample is also important given evidence that age may differentially impact these measures of brain structure.

The current study was designed to address this issue. Based on research suggesting that the prefrontal cortex is particularly vulnerable to age effects, we were primarily interested in the impact of age on prefrontal surface area and cortical thickness in healthy middle-aged to older adults, specifically within the DLPFC, ACC and OFC. We also performed an exploratory vertex-wise analysis, in which surface area and thickness are mapped onto each vertex of the cortical surface. This procedure provides an examination of the entire cortical surface rather than being limited to predefined regions of interest (ROIs). Thus, we addressed a gap in the literature by distinguishing between the two constituent components of brain volume and by examining age effects starting in midlife, an age for which less data are available in the literature. We predicted that older age would be associated with reductions in both surface area and cortical thickness in the DLPFC, ACC, and OFC.

## Materials and Methods

### Participants

Forty-six healthy, community-dwelling adults ranging in age from 51 to 81 years (*mean* = 68.54 ± 7.43; 67.39% female) participated in this study. The current study involved secondary analysis of data from healthy control participants in a larger investigation that focused on age differences in depression-related changes in brain structure and function. The larger study included functional magnetic resonance imaging (fMRI), thus, participants were required to be right-handed, speak English as their first language, and have at least 10 years of education (range = 10–20 years; *mean* = 15.05 ± 2.48). Potential participants were excluded if they scored less than 30 on the Telephone Interview for Cognitive Status (TICS; Brandt et al., 1988), the traditional cutoff for possible dementia, or if they met criteria for major psychiatric disorder based on administration of the Structured Clinical Interview for DSM-IV-TR Axis I Disorders, Research Version (First et al., 2001). Additional exclusionary criteria based on self-report included severe or acute medical illness, history of neurological conditions (e.g., stroke, head injury, epilepsy), learning disorders, and MRI contraindications. The study protocol was approved by the University of Florida’s Institutional Review Board, and all participants provided both written and verbal informed consent in accordance with the Declaration of Helsinki.

### MRI Data Acquisition

Scanning took place at the University of Florida’s McKnight Brain Institute in a 3-Tesla Phillips (Amsterdam, Netherlands) scanner. A standard 8-channel head radio-frequency coil was used to collect a high-resolution, T_1_-weighted turbo field echo structural MRI scan. Scanning parameters were as follows: TR = 28.1 ms, TE = 2.7 ms, FOV = 240 mm × 240 mm (AP) × 170 mm (RL), matrix = 240 × 240, 170 slices acquired in a sagittal orientation, flip angle = 8°, 1 mm cubic resolution (isotropic).

### Regional Surface Area and Cortical Thickness Measurement

Freesurfer version 5.3 was used to extract surface area and cortical thickness. This image processing suite provides automated parcellation of both cortical and subcortical brain structures. Details of the procedure are documented elsewhere (Fischl et al., 2002) and are freely available for download (http://surfer.nmr.mgh.harvard.edu/). In brief, processing included motion correction and averaging of T1 weighted images (Reuter et al., 2010), skull stripping using a hybrid watershed/surface deformation procedure (Ségonne et al., 2004), automated Talairach transformation, segmentation of the subcortical white matter and deep gray matter volumetric structures (Fischl et al., 2002, 2004a), intensity inhomogeneity correction (Sled et al., 1998), gray/white matter boundary tessellation, topology correction (Fischl et al., 2001; Ségonne et al., 2007), and surface deformation following intensity gradients to ensure optimal gray/white and gray/cerebrospinal fluid boarder (Dale and Sereno, 1993; Sled et al., 1998; Dale et al., 1999; Fischl and Dale, 2000; Fischl et al., 2004a; Ségonne et al., 2004, 2007). Cortical regions were parcellated into specific regions with respect to sulcal and gyral structures (Fischl et al., 2004b; Desikan et al., 2006), with manual inspection for errors in the automatic program by one of two trained raters.

Surface area was calculated along the white matter surface boundary. This surface was then tessellated and the value for each ROI represented the sum of the area of all of the triangles that compose each cortical parcellation. Interclass correlation coefficients (ICCs) were calculated for manual adjustments in surface area using a two-way mixed effects model. ICC between raters was high (≥0.876), likely reflecting the minimal manual adjustments needed following the automatic processing.

Both intensity and continuity information in segmentation and deformation procedures were used to produce representations of cortical thickness, calculated as the closest distance from the gray/white boundary to the gray/CSF boundary at each vertex on the tessellated surface (Fischl and Dale, 2000). Automated cortical thickness measures have been validated against histological analysis (Rosas et al., 2002) and manual measurements (Kuperberg et al., 2003; Salat et al., 2004). ICC for manual adjustments in cortical thickness segmentation was also high (≥0.882).

Based on *a priori* hypotheses, primary analyses focused on surface area and cortical thickness in ROIs in the DLPFC (sum of the inferior and middle frontal gyri), ACC (sum of the rostral and caudal ACC), and OFC (sum of the medial and lateral OFC). For cortical thickness, the weighted means (regional thickness divided by regional volume) were summed to create the ROIs. Additionally, exploratory analyses used a vertex-wise approach. For each subject, a thickness measurement was mapped on each vertex of the cortical surface, allowing an examination of surface area and cortical thickness across the brain. Images were smoothed using a Gaussian kernel with a full-width-half-maximum of 10 mm.

### Statistical Analyses

Bivariate correlations between surface area and cortical thickness in each ROI were calculated using Pearson correlations. To address our primary hypotheses, regression analyses were performed using SAS version 9.4 (Cary, NC, USA) with a continuous measure of age as the independent variable, and with either surface area or thickness in each ROI as the dependent variable. Analyses were performed separately by hemisphere given evidence of asymmetries in age-related brain changes (Raz et al., 1997). Analysis of surface area included a covariate for estimated total intracranial volume, and analysis of cortical thickness included a covariate for mean cortical thickness. Initially, each model also controlled for sex and education, but these variables were removed since they were not significant in any of the models. The type I error used for statistical significance was *α* ≤ 0.05 for all analyses. For the exploratory vertex-wise analyses, separate general linear models were conducted in Freesurfer with age predicting either surface area or cortical thickness at each vertex. A false discovery rate (FDR) threshold of ≤0.05 was used to account for type I error.

## Results

**Figure 1:**
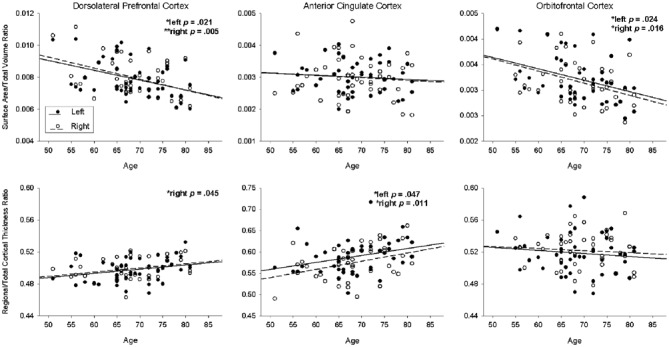
**Association of age with surface area (upper row; ratio of regional surface area to total volume) and cortical thickness (lower row; ratio of regional thickness to mean thickness) for each region of interest.** The solid line and black circles represent the left hemisphere; the dotted line and white circles represent the right hemisphere.

Surface area and cortical thickness were significantly negatively correlated in the left DLPFC (*r* = −0.329, *p* = 0.026), bilateral ACC (left *r* = −0.350, *p* = 0.017; right* r* = −0.354, *p* = 0.016), and bilateral OFC (left *r* = −0.406, *p* = 0.005; right* r* = −0.368, *p* = 0.012). Results of the regression analyses on primary ROIs are summarized in Table 1 and Figure 1. After controlling for total intracranial volume, older age was associated with decreased surface area in the bilateral DLPFC (left *B* = −47.313, *p* = 0.021, *R*^2^ = 0.310; right *B* = −57.161, *p* = 0.005, *R*^2^ = 0.356) and bilateral OFC (left *B* = −15.452, *p* = 0.021, *R*^2^ = 0.322; right *B* = −17.398, *p* = 0.021, *R*^2^ = 0.331), but not with surface area in the ACC. In contrast, cortical thickness analyses revealed greater cortical thickness at older ages in the right DLPFC (*B* = 0.002, *p* = 0.021, *R*^2^ = 0.768) and bilateral ACC (left *B* = 0.007, *p* = 0.021, *R*^2^ = 0.269; right *B* = 0.009, *p* = 0.011, *R*^2^ = 0.407) after controlling for mean thickness, while age was not associated with cortical thickness in the OFC.

**Table 1:** **Age effects on surface area and cortical thickness in the ROI analysis, adjusted for total intracranial volume (surface area) and thickness (cortical thickness)**.

	Surface area (mm^2^)		Cortical thickness (mm)	
Region	*B*	*SE (df)*	*B*	*SE (df)*
Dorsolateral prefrontal cortex
Left	−47.313*	19.668 (43)	0.002	0.001 (43)
Right	−57.161**	19.073 (43)	0.002*	0.001 (43)
Anterior cingulate cortex
Left	3.023	11.314 (43)	0.007*	0.004 (43)
Right	1.554	15.036 (43)	0.009*	0.003 (43)
Orbitofrontal cortex
Left	−15.451*	6.579 (43)	−0.002	0.002 (43)
Right	−17.398*	6.954 (43)	−0.001	0.002 (43)

Note: ROI, regional of interest; B, beta weight; SE, standard error; df, degrees of freedom; *p < 0.05, **p < 0.01.

**Figure 2:**
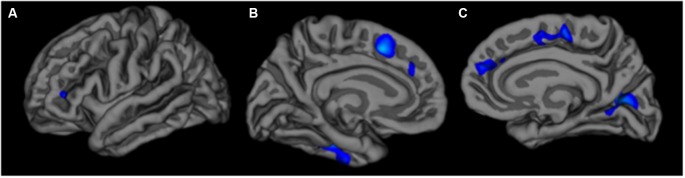
**Left lateral (A) left medial (B) and right medial (C) views of associations between older age and reduced surface area in the vertex-wise analysis.** The color scale is represented as *log (p)*.

The vertex-wise analysis revealed significantly smaller surface area at older ages in primarily frontal regions, including bilateral superior frontal, left rostral middle frontal, and right lateral orbitofrontal regions. There were no significant associations between age and cortical thickness in the vertex-wise analysis. Significant effects for the surface area analysis are summarized in Table 2 and Figure 2.

**Table 2:** **Brain regions showing a significant age effect on surface area in the vertex-wise analysis**.

		Coordinate		
Region	Side	*x*	*y*	*z*	Cluster size (mm^2^)	*p*
Cuneus	R	18.8	−65.3	12.4	624.48	0.00001
Inferior temporal	L	−45.1	−26.7	−23.3	487.44	0.00003
Superior frontal	R	7.4	−6.1	56.4	374.79	0.00007
	R	11.0	54.0	11.0	241.28	0.00013
	R	13.1	39.5	21.8	29.47	0.00049
	L	−9.0	18.3	44.7	284.72	0.00001
	L	−9.1	41.3	25.6	74.85	0.00012
Rostral middle frontal	L	−38.5	41.2	3.9	85.21	0.00009
Lingual	R	8.8	−61.0	2.2	76.71	0.00014
Lateral orbitofrontal	R	28.4	49.2	−10.5	57.89	0.00038
Fusiform	R	41.6	−34.2	−20.6	19.64	0.00029
Superior temporal	L	−56.4	−11.8	1.3	17.81	0.00032
Pars orbitalis	R	38.5	37.9	−9.0	1.36	0.00070

*Note: Talairach coordinates reflect location of peak voxel within cluster. L, left; R, right*.

## Discussion

We examined age differences in prefrontal surface area and cortical thickness in a middle-aged to older adult sample. As expected, age differences were observed for each of our ROIs; however, the nature of the differences varied, as older age was associated with smaller surface area in the DLPFC and OFC, but higher cortical thickness in the DLPFC and ACC.

The finding of reduced surface area in the DLPFC and OFC at older ages is consistent with previous cross-sectional and longitudinal research documenting age-related structural changes within these regions (Raz et al., 2010; Lemaitre et al., 2012; Hogstrom et al., 2013; Persson et al., 2014). The majority of past studies have compared young and older adults, but some studies have examined age differences in later decades (Raz et al., 2010; Storsve et al., 2014). For example, orbitofrontal and lateral prefrontal volume declined over 30-month follow-up in a study of middle-aged to older adults (Raz et al., 2010), similar to a lifespan study showing declining thickness and surface area over an average of 3.5 years (Storsve et al., 2014). In our study, older age was associated with differences in OFC surface area, but not in thickness. Although there have been reports of orbitofrontal thinning in the OFC with age (Thambisetty et al., 2010), other studies have found that thinning of the OFC plateaus at older ages (Sowell et al., 2003; Fjell et al., 2009a, 2014). This has been reported both in cross-sectional studies, in which the OFC was more preserved than other frontal regions (Salat et al., 2002; Fjell et al., 2009a,b), and in longitudinal studies, in which thinning of the OFC occurred at a slower rate than most ROIs (Sowell et al., 2003). These results suggest that the impact of age on OFC thickness may be minimal within groups of middle-age to older adults, who constituted the sample for the present study.

In contrast to results for the OFC, we found that older age was associated with reduced surface area in the bilateral DLPFC but greater cortical thickness in the right DLPFC. The different direction of the relationships of age with surface area and thickness is not surprising considering evidence of a negative correlation between these two measures in regions including the prefrontal cortex (e.g., Storsve et al., 2014). We also found a negative relationship between surface area in cortical thickness in the DLPFC, ACC and OFC. Age-related inflammatory changes may explain these findings. There is evidence that inflammation is associated with reductions in gray matter volume and surface area (Jefferson et al., 2007; Marsland et al., 2015), however, the relationship between inflammation and cortical thickness may be more complex. For example, in the Religious Orders Study, inflammation was associated with cortical thinning in some regions, but cortical thickening in others (Fleischman et al., 2010), similar to other reports of a positive relationship between inflammatory markers and cortical thickness (Sörös, 2010; Krishnadas et al., 2013). Given the chronic, low-grade inflammation that increases with age (Cevenini et al., 2010; Solana et al., 2012), older age may be characterized by concomitant surface area decreases and cortical thickness increases.

Inflammation might also contribute to the association between older age and greater cortical thickness in the bilateral ACC in the present study. There is evidence to suggest that the relationship between age and thickness in the ACC is nonlinear, such that thickness of the ACC is reduced at older age until age 60, but is greater at older ages thereafter (Fjell et al., 2014). One hypothesis to explain this observation is that increased thickness of the ACC is an indication of neuroplasticity (Draganski et al., 2006; Engvig et al., 2010) given its role in allocating attentional resources (Bush et al., 2000; Posner et al., 2007; Posner, 2012), and executive control (Westlye et al., 2010). Since we would not expect neuroplasticity to increase after age 60, this explanation would not appear to fully explain the relationship. In contrast, inflammation does increase with age, and thus appears to be a more plausible cause of age-related thickness increases. Longitudinal studies will help to clarify potential mechanisms. The lack of age-related surface area reduction in the ACC is in accordance with previous cross-sectional studies (Fjell et al., 2009a, 2014), as well as a recent longitudinal study of healthy adults in which decelerating changes across the lifespan were observed in some regions of the prefrontal cortex (Storsve et al., 2014). In contrast, other studies have documented age-related differences and decline in these regions (Raz et al., 2005, 2010). For example, older age was associated with smaller volumes in the ACC in a recent investigation of middle-aged to older adults (Hafkemeijer et al., 2014).

Variability in findings across studies of age and brain structure may be due, in part, to differences in the study samples. For example, men and women have been found to have different trajectories of age-related structural changes (Murphy et al., 1996; Coffey et al., 1998; van Velsen et al., 2013). Demographic and clinical variables such as education, vascular disease, and genetic variation may also moderate the relationships of age with brain structure (Decarli et al., 1999; Swan et al., 2000; Seshadri et al., 2004; Raz et al., 2005, 2007b; Luders et al., 2006; Brundel et al., 2010; Debette et al., 2011; Leritz et al., 2011; Villeneuve et al., 2014; Gonzalez et al., 2015) but have not always been accounted for in previous studies. Additionally, findings from cross-sectional studies may differ from longitudinal studies, as there is evidence that some regions show no cross-sectional differences but do change over time (Fjell et al., 2014).

The present study should be considered within the context of the relatively small sample size, which, together with the lack of young adults in the sample, limited our ability to directly examine nonlinear age effects. The sample size might also explain the discrepancy between the ROI analyses, which revealed a significant association between age and cortical thickness, and the exploratory vertex-wise analyses, which revealed no significant associations. The vertex-wise analyses were exploratory in nature and involved a large number of analyses that included each vertex in the cortex, thus a very strict correction for multiple comparisons was made. In our fairly small sample, small but significant effects are more difficult to detect with such a correction but are apparent in focused, hypothesis-driven ROI analyses. Nonetheless, our results are consistent with previous demonstrations of age-related differences in frontal lobe structure, and contribute to a relatively small body of literature examining age effects in middle-age to older adults. Our findings also highlight the dissociation between measures of surface area and cortical thickness. Though some studies have shown similar age effects across different structural methods (Lemaitre et al., 2012), these two measurements are dissociable metrics that are confounded in studies of gray matter volume. Some studies have found either no correlation or a weak correlation between thickness and surface area (Winkler et al., 2010; Hogstrom et al., 2013), consistent with evidence that thickness and area are genetically independent and are affected by distinct neurobiological factors, such as myelin growth, during development (Panizzon et al., 2009; Rakic et al., 2009; Rakic, 2009; White et al., 2010). Additional research is needed to clarify the distinct causes of surface area and cortical thickness changes in late life, and to determine whether or not these two measures are differentially related to clinical and functional outcomes.

Overall, the present findings suggest that reductions in surface area and greater cortical thickness in prefrontal regions occur during the later decades of life. Thus, our findings suggest aging differentially affects changes in regional surface area and cortical thickness. Longitudinal studies are needed in middle-age and older adults to confirm these findings.

## Author Contributions

AJW and VMD designed the study. VMD, SMS, CNS, JWK, MLG, SDA and TMM acquired the data. SMS, MEM, AO, and AJW processed the neuroimaging data. VMD performed the statistical analyses. All authors contributed to data interpretation and writing of the article.

## Conflict of Interest Statement

The authors declare that the research was conducted in the absence of any commercial or financial relationships that could be construed as a potential conflict of interest.

## References

[B1] Allen J. S. Bruss J. Brown C. K. Damasio H. (2005). Normal neuroanatomical variation due to age: the major lobes and a parcellation of the temporal region. Neurobiol. Aging 26, 1245–1260. 10.1016/j.neurobiolaging.2005.05.023 16046030

[B2] Brandt J. Spencer M. Folstein M. (1988). The telephone interview for cognitive status. Neuropsychiatry Neuropsychol. Behav. Neurol. 1, 111–117.

[B3] Brundel M. van Den Heuvel M. de Bresser J. Kappelle L. J. Biessels G. J. Utrecht Diabetic Encephalopathy Study G. (2010). Cerebral cortical thickness in patients with type 2 diabetes. J. Neurol. Sci. 299, 126–130. 10.1016/j.jns.2010.08.048 20869085

[B4] Bush G. Luu P. Posner M. I. (2000). Cognitive and emotional influences in anterior cingulate cortex. Trends Cogn. Sci. 4, 215–222. 10.1016/s1364-6613(00)01483-2 10827444

[B5] Cevenini E. Caruso C. Candore G. Capri M. Nuzzo D. Duro G.. (2010). Age-related inflammation: the contribution of different organs, tissues and systems. How to face it for therapeutic approaches. Curr. Pharm. Des. 16, 609–618. 10.2174/138161210790883840 20388071

[B6] Coffey C. E. Lucke J. F. Saxton J. A. Ratcliff G. Unitas L. J. Billig B.. (1998). Sex differences in brain aging: a quantitative magnetic resonance imaging study. Arch. Neurol. 55, 169–179. 10.1001/archneur.55.2.169 9482358

[B7] Curiati P. K. Tamashiro J. H. Squarzoni P. Duran F. L. Santos L. C. Wajngarten M.. (2009). Brain structural variability due to aging and gender in cognitively healthy elders: results from the Sao Paulo ageing and health study. AJNR Am. J. Neuroradiol. 30, 1850–1856. 10.3174/ajnr.A1727 19661175 PMC7051289

[B8] Dale A. M. Fischl B. Sereno M. I. (1999). Cortical surface-based analysis. I. Segmentation and surface reconstruction. Neuroimage 9, 179–194. 10.1006/nimg.1998.0395 9931268

[B9] Dale A. M. Sereno M. I. (1993). Improved localizadon of cortical activity by combining EEG and MEG with MRI cortical surface reconstruction: a linear approach. J. Cogn. Neurosci. 5, 162–176. 10.1162/jocn.1993.5.2.162 23972151

[B10] Debette S. Seshadri S. Beiser A. Au R. Himali J. J. Palumbo C.. (2011). Midlife vascular risk factor exposure accelerates structural brain aging and cognitive decline. Neurology 77, 461–468. 10.1212/WNL.0b013e318227b227 21810696 PMC3146307

[B11] Decarli C. Miller B. L. Swan G. E. Reed T. Wolf P. A. Garner J.. (1999). Predictors of brain morphology for the men of the NHLBI twin study. Stroke 30, 529–536. 10.1161/01.str.30.3.529 10066847

[B12] Desikan R. S. Ségonne F. Fischl B. Quinn B. T. Dickerson B. C. Blacker D.. (2006). An automated labeling system for subdividing the human cerebral cortex on MRI scans into gyral based regions of interest. Neuroimage 31, 968–980. 10.1016/j.neuroimage.2006.01.021 16530430

[B13] Draganski B. Gaser C. Kempermann G. Kuhn H. G. Winkler J. Büchel C.. (2006). Temporal and spatial dynamics of brain structure changes during extensive learning. J. Neurosci. 26, 6314–6317. 10.1523/jneurosci.4628-05.2006 16763039 PMC6675198

[B14] Driscoll I. Davatzikos C. An Y. Wu X. Shen D. Kraut M.. (2009). Longitudinal pattern of regional brain volume change differentiates normal aging from MCI. Neurology 72, 1906–1913. 10.1212/WNL.0b013e3181a82634 19487648 PMC2690968

[B15] Du A. T. Schuff N. Kramer J. H. Rosen H. J. Gorno-Tempini M. L. Rankin K.. (2007). Different regional patterns of cortical thinning in Alzheimer’s disease and frontotemporal dementia. Brain 130, 1159–1166. 10.1093/brain/awm016 17353226 PMC1853284

[B16] Engvig A. Fjell A. M. Westlye L. T. Moberget T. Sundseth O. Larsen V. A.. (2010). Effects of memory training on cortical thickness in the elderly. Neuroimage 52, 1667–1676. 10.1016/j.neuroimage.2010.05.041 20580844

[B17] First M. B. Gibbon M. Spitzer R. L. Williams J. B. (2001). User’s Guide for the Structured Clinical Interview for DSM-IV-TR Axis I Disorders SCID-I : Research Version. New York, NY: Biometrics Research.

[B18] Fischl B. Dale A. M. (2000). Measuring the thickness of the human cerebral cortex from magnetic resonance images. Proc. Natl. Acad. Sci. U S A 97, 11050–11055. 10.1073/pnas.200033797 10984517 PMC27146

[B19] Fischl B. Liu A. Dale A. M. (2001). Automated manifold surgery: constructing geometrically accurate and topologically correct models of the human cerebral cortex. IEEE Trans. Med. Imaging 20, 70–80. 10.1109/42.906426 11293693

[B20] Fischl B. Salat D. H. Busa E. Albert M. Dieterich M. Haselgrove C.. (2002). Whole brain segmentation: automated labeling of neuroanatomical structures in the human brain. Neuron 33, 341–355. 10.1016/S0896-6273(02)00569-X 11832223

[B21] Fischl B. Salat D. H. van der Kouwe A. J. Makris N. Ségonne F. Quinn B. T.. (2004a). Sequence-independent segmentation of magnetic resonance images. Neuroimage 23, S69–S84. 10.1016/j.neuroimage.2004.07.016 15501102

[B22] Fischl B. van der Kouwe A. Destrieux C. Halgren E. Ségonne F. Salat D. H.. (2004b). Automatically parcellating the human cerebral cortex. Cereb. Cortex 14, 11–22. 10.1093/cercor/bhg087 14654453

[B23] Fjell A. M. Walhovd K. B. Fennema-Notestine C. McEvoy L. K. Hagler D. J. Holland D.. (2009a). One-year brain atrophy evident in healthy aging. J. Neurosci. 29, 15223–15231. 10.1523/JNEUROSCI.3252-09.2009 19955375 PMC2827793

[B25] Fjell A. M. Westlye L. T. Amlien I. Espeseth T. Reinvang I. Raz N.. (2009b). High consistency of regional cortical thinning in aging across multiple samples. Cereb. Cortex 19, 2001–2012. 10.1093/cercor/bhn232 19150922 PMC2733683

[B24] Fjell A. M. Walhovd K. B. Reinvang I. Lundervold A. Salat D. Quinn B. T.. (2006). Selective increase of cortical thickness in high-performing elderly–structural indices of optimal cognitive aging. Neuroimage 29, 984–994. 10.1016/j.neuroimage.2005.08.007 16176876

[B26] Fjell A. M. Westlye L. T. Grydeland H. Amlien I. Espeseth T. Reinvang I.. (2014). Accelerating cortical thinning: unique to dementia or universal in aging? Cereb. Cortex 24, 919–934. 10.1093/cercor/bhs379 23236213 PMC3948495

[B27] Fleischman D. A. Arfanakis K. Kelly J. F. Rajendran N. Buchman A. S. Morris M. C.. (2010). Regional brain cortical thinning and systemic inflammation in older persons without dementia. J. Am. Geriatr. Soc. 58, 1823–1825. 10.1111/j.1532-5415.2010.03049.x 20863359 PMC2945260

[B28] Gonzalez C. E. Pacheco J. Beason-Held L. L. Resnick S. M. (2015). Longitudinal changes in cortical thinning associated with hypertension. J. Hypertens. 33, 1242–1248. 10.1097/hjh.0000000000000531 25693060 PMC5912213

[B29] Hafkemeijer A. Altmann-Schneider I. de Craen A. J. Slagboom P. E. van der Grond J. Rombouts S. A. (2014). Associations between age and gray matter volume in anatomical brain networks in middle-aged to older adults. Aging Cell 13, 1068–1074. 10.1111/acel.12271 25257192 PMC4326918

[B30] Hogstrom L. J. Westlye L. T. Walhovd K. B. Fjell A. M. (2013). The structure of the cerebral cortex across adult life: age-related patterns of surface area, thickness and gyrification. Cereb. Cortex 23, 2521–2530. 10.1093/cercor/bhs231 22892423

[B31] Jefferson A. L. Massaro J. M. Wolf P. A. Seshadri S. Au R. Vasan R. S.. (2007). Inflammatory biomarkers are associated with total brain volume: the framingham heart study. Neurology 68, 1032–1038. 10.1212/01.wnl.0000257815.20548.df 17389308 PMC2758770

[B32] Kabani N. Le Goualher G. MaCdonald D. Evans A. C. (2001). Measurement of cortical thickness using an automated 3-D algorithm: a validation study. Neuroimage 13, 375–380. 10.1006/nimg.2000.0652 11162277

[B33] Krishnadas R. McLean J. Batty D. G. Burns H. Deans K. A. Ford I.. (2013). Cardio-metabolic risk factors and cortical thickness in a neurologically healthy male population: results from the psychological, social and biological determinants of ill health (pSoBid) study. Neuroimage Clin. 2, 646–657. 10.1016/j.nicl.2013.04.012 24179815 PMC3777783

[B34] Kuperberg G. R. Broome M. R. McGuire P. K. David A. S. Eddy M. Ozawa F.. (2003). Regionally localized thinning of the cerebral cortex in schizophrenia. Arch. Gen. Psychiatry 60, 878–888. 10.1001/archpsyc.60.9.878 12963669

[B35] Lemaitre H. Goldman A. L. Sambataro F. Verchinski B. A. Meyer-Lindenberg A. Weinberger D. R.. (2012). Normal age-related brain morphometric changes: nonuniformity across cortical thickness, surface area and gray matter volume? Neurobiol. Aging 33, 617.e1–617.e9. 10.1016/j.neurobiolaging.2010.07.013 20739099 PMC3026893

[B36] Leritz E. C. Salat D. H. Williams V. J. Schnyer D. M. Rudolph J. L. Lipsitz L.. (2011). Thickness of the human cerebral cortex is associated with metrics of cerebrovascular health in a normative sample of community dwelling older adults. Neuroimage 54, 2659–2671. 10.1016/j.neuroimage.2010.10.050 21035552 PMC3026290

[B37] Liu R. S. Lemieux L. Bell G. S. Sisodiya S. M. Shorvon S. D. Sander J. W.. (2003). A longitudinal study of brain morphometrics using quantitative magnetic resonance imaging and difference image analysis. Neuroimage 20, 22–33. 10.1016/s1053-8119(03)00219-2 14527567

[B38] Long X. Liao W. Jiang C. Liang D. Qiu B. Zhang L. (2012). Healthy aging: an automatic analysis of global and regional morphological alterations of human brain. Acad. Radiol. 19, 785–793. 10.1016/j.acra.2012.03.006 22503890

[B39] Luders E. Narr K. L. Thompson P. M. Rex D. E. Woods R. P. Deluca H.. (2006). Gender effects on cortical thickness and the influence of scaling. Hum. Brain Mapp. 27, 314–324. 10.1002/hbm.20187 16124013 PMC6871390

[B40] Marsland A. L. Gianaros P. J. Kuan D. C. Sheu L. K. Krajina K. Manuck S. B. (2015). Brain morphology links systemic inflammation to cognitive function in midlife adults. Brain Behav. Immun. 48, 195–204. 10.1016/j.bbi.2015.03.015 25882911 PMC4508197

[B41] Murphy D. G. DeCarli C. McIntosh A. R. Daly E. Mentis M. J. Pietrini P.. (1996). Sex differences in human brain morphometry and metabolism: an *in vivo* quantitative magnetic resonance imaging and positron emission tomography study on the effect of aging. Arch. Gen. Psychiatry 53, 585–594. 10.1001/archpsyc.1996.01830070031007 8660125

[B42] Ostby Y. Tamnes C. K. Fjell A. M. Westlye L. T. Due-Tønnessen P. Walhovd K. B. (2009). Heterogeneity in subcortical brain development: a structural magnetic resonance imaging study of brain maturation from 8 to 30 years. J. Neurosci. 29, 11772–11782. 10.1523/JNEUROSCI.1242-09.2009 19776264 PMC6666647

[B43] Panizzon M. S. Fennema-Notestine C. Eyler L. T. Jernigan T. L. Prom-Wormley E. Neale M.. (2009). Distinct genetic influences on cortical surface area and cortical thickness. Cereb. Cortex 19, 2728–2735. 10.1093/cercor/bhp026 19299253 PMC2758684

[B44] Persson N. Ghisletta P. Dahle C. L. Bender A. R. Yang Y. Yuan P.. (2014). Regional brain shrinkage over 2 years: individual differences and effects of pro-inflammatory genetic polymorphisms. Neuroimage 103, 334–348. 10.1016/j.neuroimage.2014.09.042 25264227 PMC4312187

[B45] Pfefferbaum A. Rohlfing T. Rosenbloom M. J. Chu W. Colrain I. M. Sullivan E. V. (2013). Variation in longitudinal trajectories of regional brain volumes of healthy men and women (ages 10 to 85 years) measured with atlas-based parcellation of MRI. Neuroimage 65, 176–193. 10.1016/j.neuroimage.2012.10.008 23063452 PMC3516371

[B46] Posner M. I. (2012). Imaging attention networks. Neuroimage 61, 450–456. 10.1016/j.neuroimage.2011.12.040 22227132 PMC3345293

[B47] Posner M. I. Rothbart M. K. Sheese B. E. Tang Y. (2007). The anterior cingulate gyrus and the mechanism of self-regulation. Cogn. Affect. Behav. Neurosci. 7, 391–395. 10.3758/cabn.7.4.391 18189012

[B48] Preul C. Hund-Georgiadis M. Forstmann B. U. Lohmann G. (2006). Characterization of cortical thickness and ventricular width in normal aging: a morphometric study at 3 Tesla. J. Magn. Reson. Imaging 24, 513–519. 10.1002/jmri.20665 16878302

[B49] Rakic P. (2009). Evolution of the neocortex: a perspective from developmental biology. Nat. Rev. Neurosci. 10, 724–735. 10.1038/nrn2719 19763105 PMC2913577

[B50] Rakic P. Ayoub A. E. Breunig J. J. Dominguez M. H. (2009). Decision by division: making cortical maps. Trends Neurosci. 32, 291–301. 10.1016/j.tins.2009.01.007 19380167 PMC3601545

[B51] Raz N. Ghisletta P. Rodrigue K. M. Kennedy K. M. Lindenberger U. (2010). Trajectories of brain aging in middle-aged and older adults: regional and individual differences. Neuroimage 51, 501–511. 10.1016/j.neuroimage.2010.03.020 20298790 PMC2879584

[B52] Raz N. Gunning F. M. Head D. Dupuis J. H. McQuain J. Briggs S. D.. (1997). Selective aging of the human cerebral cortex observed *in vivo*: differential vulnerability of the prefrontal gray matter. Cereb .Cortex 7, 268–282. 10.1093/cercor/7.3.268 9143446

[B53] Raz N. Lindenberger U. Rodrigue K. M. Kennedy K. M. Head D. Williamson A.. (2005). Regional brain changes in aging healthy adults: general trends, individual differences and modifiers. Cereb. Cortex 15, 1676–1689. 10.1093/cercor/bhi044 15703252

[B54] Raz N. Rodrigue K. M. (2006). Differential aging of the brain: patterns, cognitive correlates and modifiers. Neurosci. Biobehav. Rev. 30, 730–748. 10.1016/j.neubiorev.2006.07.001 16919333 PMC6601348

[B55] Raz N. Rodrigue K. M. Haacke E. M. (2007a). Brain aging and its modifiers: insights from *in vivo* neuromorphometry and susceptibility weighted imaging. Ann. N Y Acad. Sci. 1097, 84–93. 10.1196/annals.1379.018 17413014 PMC2630248

[B57] Raz N. Rodrigue K. M. Kennedy K. M. Acker J. D. (2007b). Vascular health and longitudinal changes in brain and cognition in middle-aged and older adults. Neuropsychology 21, 149–157. 10.1037/0894-4105.21.2.149 17402815

[B56] Raz N. Rodrigue K. M. Head D. Kennedy K. M. Acker J. D. (2004). Differential aging of the medial temporal lobe: a study of a 5-year change. Neurology 62, 433–438. 10.1212/01.wnl.0000106466.09835.46 14872026

[B58] Resnick S. M. Pham D. L. Kraut M. A. Zonderman A. B. Davatzikos C. (2003). Longitudinal magnetic resonance imaging studies of older adults: a shrinking brain. J. Neurosci. 23, 3295–3301. 12716936 10.1523/JNEUROSCI.23-08-03295.2003 PMC6742337

[B59] Reuter M. Rosas H. D. Fischl B. (2010). Highly accurate inverse consistent registration: a robust approach. Neuroimage 53, 1181–1196. 10.1016/j.neuroimage.2010.07.020 20637289 PMC2946852

[B60] Rosas H. D. Liu A. K. Hersch S. Glessner M. Ferrante R. J. Salat D. H.. (2002). Regional and progressive thinning of the cortical ribbon in Huntington’s disease. Neurology 58, 695–701. 10.1212/wnl.58.5.695 11889230

[B61] Salat D. H. Buckner R. L. Snyder A. Z. Greve D. N. Desikan R. S. Busa E.. (2004). Thinning of the cerebral cortex in aging. Cereb. Cortex 14, 721–730. 10.1093/cercor/bhh032 15054051

[B62] Salat D. H. Kaye J. A. Janowsky J. S. (2002). Greater orbital prefrontal volume selectively predicts worse working memory performance in older adults. Cereb. Cortex 12, 494–505. 10.1093/cercor/12.5.494 11950767

[B63] Schuff N. Tosun D. Insel P. S. Chiang G. C. Truran D. Aisen P. S.. (2012). Nonlinear time course of brain volume loss in cognitively normal and impaired elders. Neurobiol. Aging 33, 845–855. 10.1016/j.neurobiolaging.2010.07.012 20855131 PMC3032014

[B64] Ségonne F. Dale A. M. Busa E. Glessner M. Salat D. Hahn H. K.. (2004). A hybrid approach to the skull stripping problem in MRI. Neuroimage 22, 1060–1075. 10.1016/s1053-8119(04)00188-0 15219578

[B65] Ségonne F. Pacheco J. Fischl B. (2007). Geometrically accurate topology-correction of cortical surfaces using nonseparating loops. IEEE Trans. Med. Imaging 26, 518–529. 10.1109/tmi.2006.887364 17427739

[B66] Seshadri S. Wolf P. A. Beiser A. Elias M. F. Au R. Kase C. S.. (2004). Stroke risk profile, brain volume and cognitive function: the framingham offspring study. Neurology 63, 1591–1599. 10.1212/01.wnl.0000142968.22691.70 15534241

[B67] Sled J. G. Zijdenbos A. P. Evans A. C. (1998). A nonparametric method for automatic correction of intensity nonuniformity in MRI data. IEEE Trans. Med. Imaging 17, 87–97. 10.1109/42.668698 9617910

[B68] Small S. A. Nava A. S. Perera G. M. Delapaz R. Stern Y. (2000). Evaluating the function of hippocampal subregions with high-resolution MRI in Alzheimer’s disease and aging. Microsc. Res. Tech. 51, 101–108. 10.1002/1097-0029(20001001)51:1<101::aid-jemt11>3.0.co;2-h 11002358

[B69] Solana R. Tarazona R. Gayoso I. Lesur O. Dupuis G. Fulop T. (2012). Innate immunosenescence: effect of aging on cells and receptors of the innate immune system in humans. Semin. Immunol. 24, 331–341. 10.1016/j.smim.2012.04.008 22560929

[B70] Sörös P. (2010). “Increased thickness of the orbitofrontal and anterior cingulate cortex in healthy aging,” in Front. Hum. Neurosci. Conference Abstract: The 20th Annual Rotman Research Institute Conference, The frontal lobes, Toronto.

[B71] Sowell E. R. Peterson B. S. Thompson P. M. Welcome S. E. Henkenius A. L. Toga A. W. (2003). Mapping cortical change across the human life span. Nat. Neurosci. 6, 309–315. 10.1038/nn1008 12548289

[B72] Spreng R. N. Wojtowicz M. Grady C. L. (2010). Reliable differences in brain activity between young and old adults: a quantitative meta-analysis across multiple cognitive domains. Neurosci. Biobehav. Rev. 34, 1178–1194. 10.1016/j.neubiorev.2010.01.009 20109489

[B73] Storsve A. B. Fjell A. M. Tamnes C. K. Westlye L. T. Overbye K. Aasland H. W.. (2014). Differential longitudinal changes in cortical thickness, surface area and volume across the adult life span: regions of accelerating and decelerating change. J. Neurosci. 34, 8488–8498. 10.1523/JNEUROSCI.0391-14.2014 24948804 PMC6608217

[B74] Swan G. E. DeCarli C. Miller B. L. Reed T. Wolf P. A. Carmelli D. (2000). Biobehavioral characteristics of nondemented older adults with subclinical brain atrophy. Neurology 54, 2108–2114. 10.1212/wnl.54.11.2108 10851372

[B75] Taki Y. Thyreau B. Kinomura S. Sato K. Goto R. Wu K.. (2013). A longitudinal study of age- and gender-related annual rate of volume changes in regional gray matter in healthy adults. Hum. Brain Mapp. 34, 2292–2301. 10.1002/hbm.22067 22438299 PMC6870527

[B76] Thambisetty M. Wan J. Carass A. An Y. Prince J. L. Resnick S. M. (2010). Longitudinal changes in cortical thickness associated with normal aging. Neuroimage 52, 1215–1223. 10.1016/j.neuroimage.2010.04.258 20441796 PMC2910226

[B77] van Velsen E. F. Vernooij M. W. Vrooman H. A. van der Lugt A. Breteler M. M. Hofman A.. (2013). Brain cortical thickness in the general elderly population: the rotterdam scan study. Neurosci. Lett. 550, 189–194. 10.1016/j.neulet.2013.06.063 23831346

[B78] Villeneuve S. Reed B. R. Madison C. M. Wirth M. Marchant N. L. Kriger S.. (2014). Vascular risk and Abeta interact to reduce cortical thickness in AD vulnerable brain regions. Neurology 83, 40–47. 10.1212/WNL.0000000000000550 24907234 PMC4114172

[B79] Westlye L. T. Walhovd K. B. Dale A. M. Bjørnerud A. Due-Tønnessen P. Engvig A.. (2010). Life-span changes of the human brain white matter: diffusion tensor imaging (DTI) and volumetry. Cereb. Cortex 20, 2055–2068. 10.1093/cercor/bhp280 20032062

[B80] White T. Su S. Schmidt M. Kao C. Y. Sapiro G. (2010). The development of gyrification in childhood and adolescence. Brain Cogn. 72, 36–45. 10.1016/j.bandc.2009.10.009 19942335 PMC2815169

[B81] Winkler A. M. Kochunov P. Blangero J. Almasy L. Zilles K. Fox P. T.. (2010). Cortical thickness or grey matter volume? The importance of selecting the phenotype for imaging genetics studies. Neuroimage 53, 1135–1146. 10.1016/j.neuroimage.2009.12.028 20006715 PMC2891595

[B82] Ziegler G. Dahnke R. Jäncke L. Yotter R. A. May A. Gaser C. (2012). Brain structural trajectories over the adult lifespan. Hum. Brain Mapp. 33, 2377–2389. 10.1002/hbm.21374 21898677 PMC6870331

